# Controlling symmetry and localization with an artificial gauge field in a disordered quantum system

**DOI:** 10.1038/s41467-018-03481-9

**Published:** 2018-04-11

**Authors:** Clément Hainaut, Isam Manai, Jean-François Clément, Jean Claude Garreau, Pascal Szriftgiser, Gabriel Lemarié, Nicolas Cherroret, Dominique Delande, Radu Chicireanu

**Affiliations:** 1 CNRS, UMR 8523, Laboratoire de Physique des Lasers Atomes et Molécules, Université de Lille, 59000 Lille, France; 2 Laboratoire de Physique Théorique, IRSAMC, Université de Toulouse, CNRS, 31062 Toulouse, France; 3 Department of Physics, Sapienza University of Rome, P.le A. Moro 2, 00185 Rome, Italy; 4 Laboratoire Kastler Brossel, UPMC-Sorbonne Universités, CNRS, ENS-PSL Research University, Collège de France, 4 Place Jussieu, 75005 Paris, France

## Abstract

Anderson localization, the absence of diffusion in disordered media, draws its origins from the destructive interference between multiple scattering paths. The localization properties of disordered systems are expected to be dramatically sensitive to their symmetries. So far, this question has been little explored experimentally. Here we investigate the realization of an artificial gauge field in a synthetic (temporal) dimension of a disordered, periodically driven quantum system. Tuning the strength of this gauge field allows us to control the parity–time symmetry properties of the system, which we probe through the experimental observation of three symmetry-sensitive signatures of localization. The first two are the coherent backscattering, marker of weak localization, and the recently predicted coherent forward scattering, genuine interferential signature of Anderson localization. The third is the direct measurement of the *β*(*g*) scaling function in two different symmetry classes, allowing to demonstrate its universality and the one-parameter scaling hypothesis.

## Abstract (web summary)

Periodically kicked rotors are useful in exploring localization phenomenon. Here the authors use ultracold Cs atoms kicked by short laser pulses and show that the periodic modulation results into interference signals as a signature of weak and strong localizations.

## Introduction

Symmetry, disorder, and chaos are ubiquitous in both classical and quantum physics. These concepts are intimately intertwined: In a disordered crystal for instance, disorder stems from the absence of translational symmetry. But this does not mean that symmetries are absent in disordered/chaotic systems; on the contrary, they play a central role, as systems presenting the same symmetries display analogous properties. This idea led to the fundamental concept of universality class, grounding the famed random matrix theory^1^. In a dirty metal for instance, breaking the time-reversal symmetry (*T*-symmetry) has a profound effect on transport observables like electrical and thermal conductivities^2^. A common way to break the *T*-symmetry for charged particles is to apply a magnetic field. For neutral systems, where magnetic fields are inoperative, the concept of artificial gauge field^3–5^ has been introduced. It consists in building Hamiltonians that behave as if a gauge field were present. Artificial gauge fields have been realized in inhomogeneous or lattice systems, and very recently in the presence of disorder^6^.

In the present work, we exploit the simplicity and flexibility of driven cold-atom systems to generate such an artificial gauge field. For this purpose, we build on the well-known atomic kicked rotor^7^, a paradigm of both classical and quantum Hamiltonian chaos, which can be mapped onto an Anderson-like Hamiltonian in any dimension^8,9^. This system is realized experimentally by submitting laser-cooled atoms to short pulses (kicks) of a far-detuned laser standing wave.

By engineering the periodic driving, we obtain an experimental knob providing complete control of the relevant symmetry of the system, here the product of parity and time-reversal (*PT*-symmetry)^10–12^. Furthermore, the accumulated phase of a quantum particle along a closed multiple-scattering path is independent of the sense in which the loop is traveled when *PT*-invariance holds (defining the so-called orthogonal symmetry class), but not when it is broken (defining, for spinless systems, the unitary class), an effect that strongly affects quantum interference in localization phenomena. This allows us to directly observe the impact of this symmetry changing on interference signatures of localization in disordered media, and to study the universal transport properties in the two symmetry classes.

## Results

### Artificial gauge fields in disordered Floquet systems

We first show how to engineer the driving of Floquet systems to manipulate their fundamental symmetry properties. For this purpose, we consider a generalized kicked rotor Hamiltonian, to which we add a temporal dependence of the amplitude ${\cal K}(t)$ and of the spatial phase *a*(*t*) in the potential term, both periodic in time:

1 $$\hat H = \frac{{\hat p^2}}{2} + {\cal K}(t)\,\,{\mathrm{cos}}\left[ {\hat x - a(t)} \right]\mathop {\sum}\limits_n {\kern 1pt} \delta (t - n),$$

The position $\hat x$ and momentum $\hat p$ are expressed in dimensionless units, and satisfy the canonical commutation relation $[\hat x,\hat p] = i\hbar_{\mathrm{e}}$, with $\hbar_{\mathrm{e}}$ playing the role of an effective Planck’s constant (see Methods for definition of units). When ${\cal K} = {\mathrm{const}}$. and *a* = 0, we recover the standard kicked rotor, which can be mapped onto an Anderson-like tight-binding model in momentum space^7,8^ with diagonal pseudo-disorder.

When ${\cal K}(t)$ is temporally modulated at a period 2*π*/*ω*_2_ incommensurate with the kick period, it has been shown^9,13,14^ that the temporal modulation can be taken into account by adding an effective spatial coordinate *x*_2_ = *ω*_2_*t* + *φ* along a synthetic dimension labeled “2” (“1” refers to the physical dimension along which all measurements are performed). Here, we study the situation where the driving modulations have a period which is an integer multiple of the kick period (*ω*_2_ = 2*π*/*N*), i.e. ${\cal K}(t + N) = {\cal K}(t)$ and *a*(*t* + *N*) = *a*(*t*) with *N* an integer. In this case, the synthetic dimension is also periodic with twisted boundary conditions (see below). Such a system maps onto a synthetic nanotube threaded by an artificial gauge field. The flux of this artificial gauge field through the transverse section of the nanotube can be easily controlled by changing the initial phase *φ* of the temporal modulation.

**Fig. 1:**
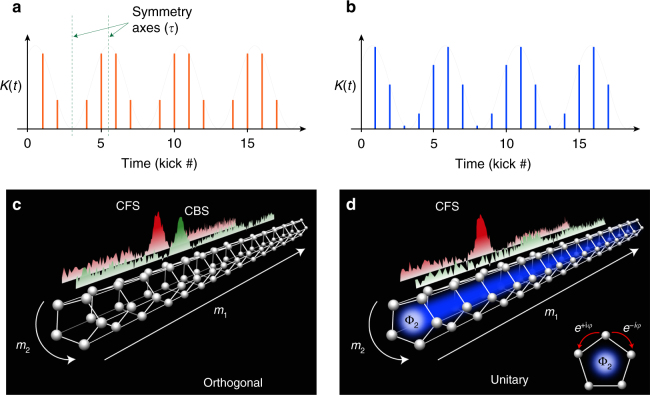
Artificial gauge fields engineering via periodically modulated driving. By tailoring the temporal dependence of the driving parameters—either the amplitude ${\cal K}(t)$ or the phase *a*(*t*)—we are able to create an artificial gauge field which controls the time-reversal symmetry properties of a periodically driven (Floquet) system. For example, for a time-symmetric kick sequence **a** the system belongs to the orthogonal symmetry class, whereas a kick sequence without any particular symmetry axes **b** puts the system in the unitary symmetry class (broken *T*-symmetry). **c**, **d** Our kicked-rotor system with periodic amplitude (or phase) modulations (1) maps on a disordered synthetic nanotube in momentum space threaded by an artificial Aharonov–Bohm flux Φ_2_. For the symmetric sequence **a** this flux is zero, whereas a non-symmetric sequence **b** corresponds to the presence of a non-zero Aharonov–Bohm flux Φ_2_ (sketched as the light blue area). Experimentally, two distinct interference signatures can be used to characterize symmetry and localization: the disappearance of the CBS peak is a clear-cut signature of the symmetry breaking, while the emergence of a CFS peak is a direct interference signature of the onset of Anderson localization, in both symmetry classes

Without loss of generality, it is convenient to illustrate the fundamental mechanism of creation and control of the artificial gauge field by using the specific example of a period-*N* amplitude modulation (see Fig. 1a, b):

2 $${\cal K}(t) = K\left[ {1 + {\mathrm{cos}}\left( {\frac{{2\pi t}}{N} + \varphi } \right)} \right]\,{\mathrm{and}}\,\,a(t) = 0.$$

The temporal dynamics of an arbitrary initial state *χ*(*x*) can be mapped on that of a two-dimensional pseudo-rotor with Hamiltonian^14^: $${\cal H} = p_1^2/2 + 2\pi p_2/N + K{\kern 1pt} {\mathrm{cos}}{\kern 1pt} x_1\left[ {1 + {\mathrm{cos}}{\kern 1pt} x_2} \right]$$$$\mathop{\sum}\nolimits_n {\kern 1pt} \delta (t - n)$$, with initial condition *χ*(*x*_1_)*δ*(*x*_2_ − *φ*). Here, “1” represents the physical dimension (*x*_1_ = *x*, *p*_1_ = *p*), and the synthetic dimension “2” is an ancillary space with 0 ≤ *x*_2_ < 2*π*, where the period-*N* dynamics is simply given by *x*_2_ = *φ* + 2*πt*/*N* (mod. 2*π*). This equivalent 2D Hamiltonian is time-periodic with period 1. Its Floquet states—eigenstates of the evolution operator over one period with eigenvalue *e*^i*ω*^—are also solutions of a tight-binding model: $$\epsilon _{\bf{m}}{\mathrm{\Psi }}_{\bf{m}} + \mathop {\sum}\nolimits_{\bf{r}} {\kern 1pt} W_{\bf{r}}{\mathrm{\Psi }}_{{\bf{m}} - {\bf{r}}} = 0$$ where **m** ≡ (*m*_1_, *m*_2_) and **r** label the sites of a 2D square lattice which correspond to momenta in units of the effective Planck’s constant $$\hbar_{\mathrm{e}}$$, and Ψ_**m**_ are the components of the Floquet quasi-states. The site energy $$\epsilon _{\bf{m}}$$ is $$\epsilon _{\bf{m}}$$ = $${\tan}\left\{ {\left[ {\omega - \left( {\hbar_{\mathrm{e}}m_1^2/2 + 2 \pi m_2/N} \right)} \right]/2} \right\}$$ and the hopping amplitudes *W*_**r**_ are coefficients of the twofold Fourier expansion of $$W({x}_{1},{x}_{2})=\tan\left[K\cos{x}_{1}(1+\cos{x}_{2})/2\hbar_{\mathrm{e}}\right]$$^9^.

When $\hbar_{\mathrm{e}}$ is incommensurate with 2*π*, the site energies constitute a pseudo-random sequence in the direction “1”, which accounts for the disordered character of our system in momentum space, leading to dynamical localization, i.e., Anderson localization in momentum space. The site energies $\epsilon _{m_1,m_2}$ form a pseudo-random set which is periodic along the direction “2” with period *N*. Thus, we can use the Bloch theorem along the direction 2 and write any Floquet state as ${\mathrm{\Psi }}_{m_1,m_2} = {\mathrm{e}}^{ - {\mathrm{i}}m_2\phi _2}{\kern 1pt} \psi _{m_1,m_2}$, where *ϕ*_2_ is the Bloch phase and $\psi _{m_1,m_2 + N} = \psi _{m_1,m_2}$ is periodic in direction 2. This is equivalent to having twisted boundary conditions along *m*_2_. The initial state $\left| {\mathrm{\Xi }} \right\rangle$ of the 2D pseudo-rotor—whose wavefunction is $\left\langle {x_1,x_2{\mathrm{|\Xi }}} \right\rangle = \chi (x_1)\delta (x_2 - \varphi )$ can be expanded in the momentum basis: $\left| {\mathrm{\Xi }} \right\rangle = \mathop {\sum}\nolimits_{m_1,m_2} {\kern 1pt} {\mathrm{\Xi }}_{m_1,m_2}\left| {m_1,m_2} \right\rangle$. The initial condition in direction 2 can be expanded in the momentum eigenbasis as: $\delta (x_2 - \varphi ) = \mathop {\sum}\nolimits_{m_2 = - \infty }^{ + \infty } {\kern 1pt} {\mathrm{e}}^{ - {\mathrm{i}}m_2\varphi }{\mathrm{e}}^{{\mathrm{i}}m_2x_2}$^14^ which implies ${\mathrm{\Xi}}_{m_1,m_2 + N} = {\mathrm{e}}^{ - {\mathrm{i}}N \varphi} {\mathrm{\Xi }}_{m_1,m_2 + N}$ Thus, the choice of the initial condition imposes the Bloch phase *ϕ*_2_ = *φ*.

The Hilbert space thus reduces to a synthetic nanotube along direction 1, with *N* sites in the transverse section along direction 2 (Fig. 1c, d). The initial phase *φ* of the temporal modulation controls the flux through the nanotube. Indeed, the Floquet eigenequation can be rewritten for the periodic function *ψ* as:

3 $$\epsilon _{m_1,m_2}\psi _{m_1,m_2} + \mathop {\sum}\limits_{r_1,r_2} {\kern 1pt} W_{r_1,r_2}{\kern 1pt} {\mathrm{e}}^{{\mathrm{i}}\varphi r_2}{\kern 1pt} \psi _{m_1 - r_1,m_2 - r_2} = 0$$

The hopping matrix elements in Eq. (3) have caught a phase *φr*_2_. This is somewhat similar to a 2D system exposed to a uniform magnetic field^15,16^. However, the geometry here is not that of a planar system, but rather a quasi-1D system or a nanotube infinite along direction 1 and with *N* transverse sites along direction 2. Indeed, a closed loop *m*_2_ = 0 → 1 → 2… → *N* − 1 → 0 will pick a total phase Φ_2_ = *Nφ*, while the counter-propagating loop will pick the opposite phase −*Nφ*. In contrast, no phase is picked along a plaquette (*m*_1_, *m*_2_) → (*m*_1_ + 1, *m*_2_) → (*m*_1_ + 1, *m*_2_ + 1) → (*m*_1_, *m*_2_ + 1) → (*m*_1_, *m*_2_). Thus, the effective gauge field flux Φ_2_ is analogous to a magnetic flux, with the magnetic field along the axis “1” of the nanotube.

If the modulation period *N* is ≥3, a generic value of *φ* corresponds to a non-vanishing (mod. *π*) flux Φ_2_. In such a situation, it is not possible to unwind all the phases in Eq. (3) and the system is expected to be in the unitary symmetry class, where all anti-unitary symmetries—product of time-reversal by a geometrical unitary operation—are broken. (The case *N* = 2 is special, as the nanotube then degenerates in a two-leg ladder with a single transverse hopping matrix element, hence the system is in the orthogonal class whatever *φ*). In contrast, if Φ_2_ = 0 (mod. *π*), all hopping terms can be made real and the system is expected to be in the orthogonal class:

4 $${N \varphi = 0} \left( {{\mathrm{mod}} {.} {\pi}} \right) \,{:} \,{\mathrm{orthogonal}}{\mathrm{class}} \\ {N \varphi \ne 0} \left( {{\mathrm{mod}} {.} {\pi}} \right) \,{:} \, {\mathrm{unitary}}{\mathrm{class}}{.}$$

This simple condition can also be deduced from a direct analysis of the kick sequences (Fig. 1a, b). For the kicked rotor (1), the relevant anti-unitary symmetry is the product of time-reversal by parity (*PT*-symmetry)^10,11^. The Hamiltonian being explicitly time-dependent, there exists a family of operators ${\cal T}_\tau$ : *t* → 2*τ* − *t*; *x* → −*x*; *p* → *p*, depending on the temporal origin of the time reversal. The condition for ${\cal T}_\tau$ to be a symmetry operation requires that the sequence of kick amplitudes ${\cal K}(t)$ be symmetric around some time *τ* (Fig. 1a). For the kick sequence in Eq. (2), it is easy to show that this happens only when *φ* is an integer multiple of *π*/*N*, in complete agreement with Eq. (4).

In the more general case of the Hamiltonian (1), it requires additionally that the kick phases *a*(*t*) be antisymmetric (as ${\cal T}_\tau$ changes *x* to −*x*). Note that, in this general case, the mapping of the system on a nanotube is slightly more complicated, but the universality class is determined by the symmetric (resp. antisymmetric) character of the kick amplitudes (resp. phases). The Hamiltonian (5) used in the next section can be mapped on a bilayer nanotube with a magnetic field, and the Hamiltonian used in the last section on a nanotube with a more complicated artificial gauge field (the details of these mappings will be published elsewhere).

### Coherent back and forward scattering

Interference phenomena, which are at the core of Anderson localization, are very sensitive to symmetry breaking. Coherent backscattering (CBS)^17^ is a simple example: a consequence of the *PT*-symmetry is that pairs of scattering paths associated with the same geometrical loop, but traveled in opposite senses, accumulate the same quantum phase and thus interfere constructively. When the symmetry is broken, these pairs of paths become out of phase and CBS disappears. However, in the presence of (strong) Anderson localization, other non-trivial quantum interference effects exist, such as the coherent forward scattering (CFS), recently predicted theoretically^18^ (see also ref. ^19^ in the context of the kicked rotor). Contrary to CBS, the CFS is insensitive to the symmetry breaking and, for unbound systems, requires the onset of Anderson localization in order to show up^20–23^. While experimental observations of CBS have been achieved in many different systems^24–29^, to the best of our knowledge no experimental observation of the CFS had been reported up till now.

In spatially disordered systems, CBS and CFS manifest in the reciprocal space as two peaks centered around −**k**^0^ (backward) and +**k**^0^ (forward) directions of the velocity distribution of a wave packet initially launched with a well-defined wave vector **k**^0^^18^. Alternatively, the constructive interference between time-reversed loops also manifests in the direct (configuration) space by an enhanced probability to return to the original position^17^.

This interference is visible, in our system, in a mixed momentum/configuration space representation (*p*_1_,*x*_2_), in which the initial state is localized. Starting with the initial conditions *p*_1_(*t* = 0) ≈ 0 and *x*_2_(*t* = 0) = +*φ*, a CBS peak should be observed around *p*_1_ = 0 at *x*_2_ = −*φ* (in the presence of the *PT*-symmetry) and a CFS peak around *p*_1_ = 0 at *x*_2_ = +*φ*^19^. Because of the time-dependence of *x*_2_(*t*) = *x*_2_(0) + 2*πt*/*N*, we thus expect to observe CBS and CFS at different times, depending on the initial phase *φ* of the modulation (see Methods). Both CBS and CFS are measurable in the physical dimension *p*_1_ as peaks around the initial momentum *p*_1_ ≈ 0. The temporal modulation is thus essential to separate them, so that they appear at different moments during the kick sequence.

We experimentally studied the CBS and CFS effects by using a thermal, ultra-cold cloud of Cs atoms kicked by a series of short pulses of a far-detuned standing wave (see Methods) created by a pair of counter-propagating laser beams. The amplitude and relative phase of the beams can be changed from one kick to another in order to create arbitrary sequences ${\cal K}(t)$ and *a*(*t*). After the application of the kicks, we perform a time-of-flight measurement of the probability density in momentum space ${\mathrm{{\Pi}}}(p_1,t) = \left| {{\mathrm{\Psi }}(p_1,t)} \right|^2$ vs. time *t*. This gives us access to the temporal dynamics of the return probability^17^, i.e. the zero-momentum probability density ${\mathrm{{\Pi}}}_0(t) = \left| {{\mathrm{\Psi }}(p_1 = 0,t)} \right|^2$. Using this observable, we are able to distinctly observe the CBS and CFS effects, and to study their dynamics and behavior with respect to the relevant symmetries of the system.

Although it is possible to observe CBS and CFS using the Hamiltonian (2) discussed above, it turns out (Methods) that the two peaks can be better separated temporally by using a combined modulation of amplitude and phase. For this reason, in our experiment we utilize the following period-10 Hamiltonian, which also belongs to the class of models (1):

5 $${\cal K}(t) = K\left[ {1 + {\mathrm{cos}}\left( {\frac{{2\pi (t - 1)}}{5}} \right)} \right]\,{\mathrm{and}}\,\, a(t) = - a,t\, {\mathrm{even}}\\ {\cal K}(t) = K\left[ {1 + {\mathrm{cos}}\left( {\frac{{2\pi (t - 1)}}{5} + \tilde \varphi } \right)} \right]\,{\mathrm{and}}\,\,a(t) = a,t\, {\mathrm{odd}}{{.}}$$

**Fig. 2:**
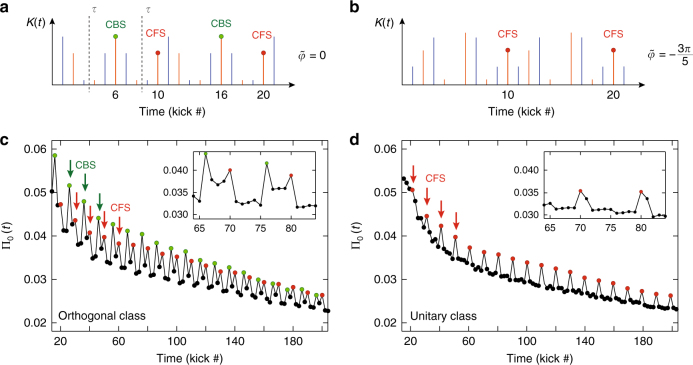
Experimental observation of CBS and CFS peaks in two symmetry classes. Using two periodically modulated kick sequences with different symmetry properties, we measure the time-evolution of the zero-momentum probability density Π_0_(*t*). **a** The kick sequence with $\tilde \varphi = 0$ in Eq. (5) has symmetric amplitudes ${\cal K}(t)$ and antisymmetric phases *a*(*t*) by reversing time around *τ* = 3.5 (and 8.5) kicks, so that the system is in the orthogonal symmetry class. **b** For $\tilde \varphi = - 3\pi {\mathrm{/}}5$, the sequence has no symmetry, putting the system in the unitary class. **c** In the orthogonal class, we observe two distinct enhancements of Π_0_(*t*), at times *t* = 6 (mod. 10) and *t* = 0 (mod. 10), associated to CBS (green) and CFS (red) peaks, respectively. The CBS peaks have maximum contrast early during the kick sequence, and decrease due to stray decoherence, whereas the CFS peaks start by slowly increasing in contrast, and equalize the CBS at longer times. This constitutes a genuine interferential signature of the emergence of Anderson localization. **d** The time evolution of Π_0_ obtained with a Hamiltonian with broken *PT*-symmetry clearly shows the disappearance of the CBS peaks in the unitary class. The CFS peaks, insensitive to the symmetry breaking, continue to be present, with a contrast following the same increasing trend at short times

As explained above, the symmetry properties of the Hamiltonian (5) are controlled by tuning the parameter $\tilde \varphi$, while the additional phase modulation *a*(*t*), with period 2, makes CBS and CFS observable only at even kicks. The CFS peak is observed at kicks multiples of the period *N* = 10 of the system: 10, 20, 30… (see Methods). The CBS peaks, on the other hand, can exist only if the Hamiltonian is *PT*-symmetric. This is the case if $\tilde \varphi$ is an integer multiple of 2*π*/5, when the kick sequence displays *PT*-symmetry axes *τ*, as illustrated in Fig. 2a. The CBS is then observed at kicks which are symmetric to the initial kick with respect to these axes, that is at times 6, 16, 26… (see Methods). In contrast, when $\tilde \varphi$ is not multiple of 2*π*/5 (see Fig. 2b) the sequence has no symmetry axis, and no CBS shows up. We thus see that a suitable choice of the symmetry properties allows for a clear temporal separation of the two peaks.

The experimental results for $\tilde \varphi = 0$ (Fig. 2c) display two characteristic features: First, the general trend of Π_0_(*t*) is a decay vs. *t*, due to the spreading of the initially narrow wave packet in momentum space. This decay slows down at long times, when localization sets in. Second, we observe pronounced peaks at kicks 20, 26, 30, 36, etc. From this series of peaks, one can however distinguish two subsequences with different properties: The CBS series at *t* = 6 (mod. 10) has a maximal contrast at the beginning, which slowly decreases with time, while the contrast of the CFS series at *t* = 0 (mod. 10) increases at short times. On a longer time scale (set by the localization time *t*_loc_), the CFS amplitude asymptotically converges toward the CBS one, and the two peaks become twins after localization has set in. This constitutes a direct interferential proof of the occurrence of Anderson localization. Adding a phase $\tilde \varphi$ to the modulation should break the *PT*-symmetry, as explained above. Indeed, this manifests (Fig. 2d) in the disappearance of the CBS peaks at *t* = 6 (mod. 10), whereas at pulses *t* = 0 (mod. 10) the CFS peaks survive and follow the increasing trend, until saturating at *t* ~ *t*_loc_.

**Fig. 3:**
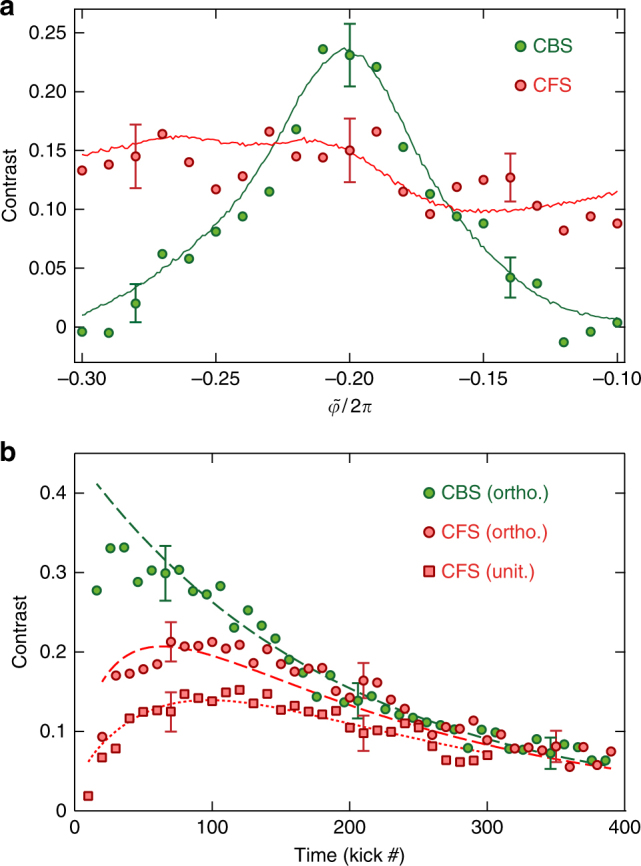
Symmetry-breaking and temporal characteristics of the CBS and CFS peaks. **a** The experimental CBS (green) and CFS (red) contrasts were measured vs. the parameter $\tilde \varphi$, which controls the symmetry class. The data are taken at *t* = 70 kicks, when the CFS contrast approaches that of the CBS. The CBS contrast is maximum at $\tilde \varphi = - 2\pi {\mathrm{/}}5$, where there is a perfect *PT*-symmetry. When $\tilde \varphi$ varies, the CBS contrast decreases, and eventually vanishes when the symmetry is completely broken. Contrary to CBS, the CFS contrast is almost insensitive to the value of $\tilde \varphi$. The error bars indicate the typical experimental uncertainty. The solid lines are ab initio numerical simulations^33^ using experimentally measured parameters. **b** The time evolutions of the CBS (orthogonal, green) and CFS (orthogonal—red circles, and unitary—red squares) contrasts corresponding to Fig. 2. The CBS follows an exponential decay (dashed green line, fit), due to decoherence, with a fitted time constant *t*_dec_ ≈ 190. The CFS contrasts are fitted using the equations in refs. ^22^ and^35^ (red lines) with decoherence effects included. This yields *t*_loc_ ≈ 40 in the unitary class and *t*_loc_ ≈ 37 in the orthogonal class

To test their dependence on $\tilde \varphi$, we monitor the contrasts of the CBS and CFS peaks (see the Methods section for details on contrast definition and measurement procedure). The results are shown in Fig. 3a: at $\tilde \varphi = - 2\pi /5$ (which preserves the *PT*-symmetry) we observe a pronounced maximum of contrast for the CBS peaks, present here at kicks 2 (mod. 10) (see Methods). The decrease of the CBS contrast around this value is a clear signature of the symmetry breaking (an analogous control, albeit not breaking the symmetry, was developed in refs. ^30,31^ for CBS in spatially disordered ultracold experiments). Although the geometry is not strictly identical, this is qualitatively similar to the magneto-resistance effect^32^ induced in a solid-state sample when time-reversal symmetry is broken by an external magnetic field. On the other hand, the contrast of the CFS peak is insensitive to $\tilde \varphi$, showing its robustness vs. the *PT*-symmetry breaking. These effects are well reproduced by numerical simulations of wave packet evolution (solid lines in Fig. 3a) based on a Fourier transform method^33^.

The time-dynamics of the CFS contrast has been theoretically predicted in ref. ^22^, using a non-perturbative, fully time-resolved analytical description of a quantum quench in an Anderson-localized unitary system. Unlike the CBS peak, which is present at short times with maximal contrast, the CFS peak requires (strong) Anderson localization in order to show up, on a time scale set by the localization time *t*_loc_.

In our experiment (Fig. 3b), the slow decay of both peaks at longer times is due to stray decoherence. The CBS contrast follows an exponential decay *C*_*B*_(*t*) = *C*_0_ exp(−*t*/*t*_dec_)^34^ and is an excellent benchmark for decoherence in our system. A fit gives the decoherence time *t*_dec_ ≈ 190 and the initial amplitude of the CBS contrast *C*_0_ ≈ 0.45 (lower than unity, due to a finite initial momentum width effect). In the unitary class, the CFS dynamics is very well fitted by the analytical formula of ref. ^22^ multiplied by the same exponential decay due to decoherence: *C*_*F*_(*t*) = *C*_0_*I*_0_(2*t*_loc_/*t*)exp(−2*t*_loc_/*t*)exp(−*t*/*t*_dec_) with *t*_loc_ ≈ 40 the only fitting parameter (*I*_*ν*_ is the modified Bessel function of order *ν*). Very recently, an analytical formula for the CFS dynamics in the orthogonal class has been given in ref. ^35^, which, when including decoherence effects, gives: *C*_*F*_(*t*) = *C*_0_[*I*_0_(2*t*_loc_/*t*) + *I*_1_(2*t*_loc_/*t*)]exp(−2*t*_loc_/*t*)exp(−*t*/*t*_dec_). A one-parameter fit using this expression is found to be in very good agreement with our experimental data, and gives *t*_loc_ ≈ 37. Our measurements clearly confirm that the CFS contrast dynamics is faster in the Orthogonal class, because of the presence of simple loops favoring Anderson localization on a shorter time scale.

These observations demonstrate that the CFS is a marker of the non-trivial quantum interferences needed to build Anderson localization in quantum disordered systems. The fact that we can observe a destruction of CBS in the presence of a surviving CFS is a clear-cut proof of the *PT*-symmetry breaking, and that other effects, such as decoherence, are not at stake for the destruction of the CBS (Methods). Hence, this represents an unambiguous evidence of the changing of our system from the orthogonal to the unitary class under the effect of the artificial gauge field.

### Symmetry and transport—universal one-parameter scaling law

The interference phenomena leading to Anderson localization also dramatically influence the bulk transport in disordered quantum systems. First corrections to the classical (incoherent) diffusion coefficient *D*_0_, known as weak localization, come from CBS-type interferences which enhance the return probability of a quantum particle^17^. This quantum corrections are directly linked to the presence of the *PT*-symmetry. In absence of this symmetry, more complex CFS-type interferences induce a slower deviation from diffusive behavior, with a distinct form.

An instrumental progress in the theory of metal-insulator transitions was the so-called one-parameter scaling theory introduced by Abrahams et al.^36^. It shows that, irrespective of the microscopic details of the system, transport properties should obey a universal scaling behavior, characterized by a single quantity, $\beta\equiv {\mathrm {d}}\ln (g)/{\mathrm{d}}\ln (L)$, the logarithmic derivative of the dimensionless conductivity *g* with respect to the size *L* of the system, which is a measure of transport. Expressed only as a function of the conductivity *g* itself, the resulting *β*(*g*) function is universal, that is, independent of microscopic details. This function has played a central role in the study of disordered systems. Based on the momentum spreading of a kicked wave packet, we here present a direct and simple experimental measurement of the *β*(*g*) scaling function, for both orthogonal and unitary classes, as well as a test of its universality within each symmetry class.

As it maps on a pseudo-random Anderson model (see above), the kicked rotor should obey a one-parameter scaling law. It is however a dynamical system, so that one has to build dynamical quantities—which are the equivalent of the system size *L* and the dimensionless conductance *g*. The natural choice for the system size *L* is the number of lattice sites effectively populated. In momentum space, the lattice sites are momentum eigenstates separated by $\Delta p=\hbar_{\mathrm{e}}$, so that we can define $L = \sqrt {\left\langle {p_1^2}(t)\right\rangle}/\hbar_{\mathrm{e}}$. Following ref.^37^, we also define $g = N\sqrt {\left\langle {p_1^2(t)} \right\rangle } /t \hbar_{\mathrm{e}}$ as the effective conductance. In the classical regime where the dynamics is diffusive, this leads to $g=2N D_{0}/\hbar_{\mathrm{e}} L$, a perfectly reasonable result, as the conductance decreases like the inverse of the system size (Ohm’s law) and is proportional to the diffusion coefficient $D_0$ (Einstein’s law). The prefactor *N* takes into account the fact that our synthetic quasi-1D system consists of *N* transverse channels (see above), so that its conductance is *N* times larger than for a purely 1D system. Note that this definition immediately leads to *β* = −1, as expected for a classical diffusive quasi-1D system.

As a logarithmic derivative, the *β*(*g*) function is extremely sensitive to experimental noise. A proper estimation of *β*(*g*) also requires to average over a sufficiently large number of disorder realizations, chosen accordingly with the desired *PT*-symmetry properties defining the universality class. Achieving this goal is not possible with the Hamiltonian (5), which does not provide enough degrees of freedom. In the frame of Hamiltonian (1), the best strategy turns out to be an average over a periodic series of *N* randomly chosen phases ${\cal A}_N = \left\{ {a_1,a_2, \ldots ,a_N} \right\}$ i.i.d. in [0, 2*π*], with ${\cal K}(t) = K = {\mathrm{const}}$. Then, the ${\cal A}_N$ series is *PT*-symmetric if *a*_*k*_ = −*a*_*N*−*k*+1_, ∀*k* integer (1 ≤ *k* ≤ *N*).

**Fig. 4:**
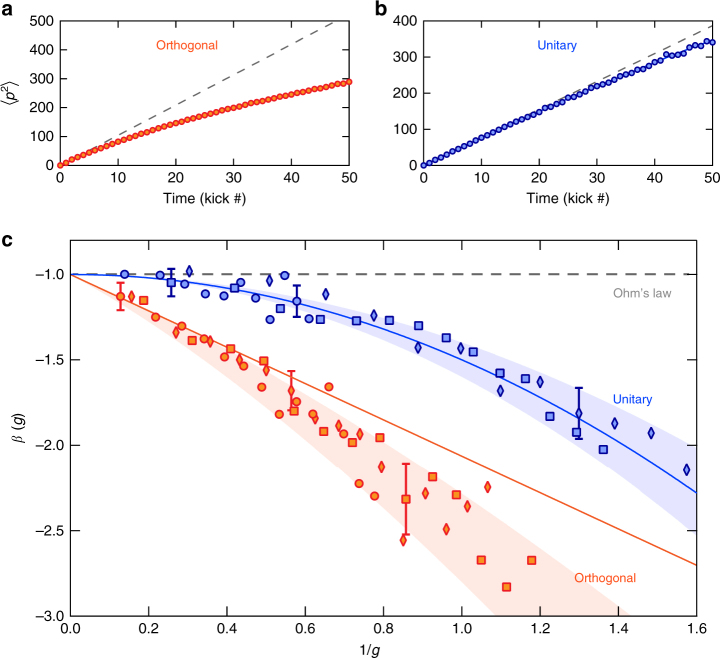
Symmetry-class dependence of the one-parameter scaling function *β*(*g*). **a**, **b** Time-evolution of $\left\langle {p_1^2} \right\rangle$ in the weak-localization regime in the two symmetry classes. In the orthogonal class (**a**), closed-loop corrections lead to a rapid deviation from classical diffusion (dashed line). In the unitary class (**b**), these corrections are absent, which qualitatively translates in a much slower departure from classical diffusion. In both cases, *D*_0_ is the same within ~20%. **c** Experimental dependence of the *β*(*g*) function on the dimensionless conductance $g = N\sqrt {\left\langle {p_1^2} \right\rangle } {\mathrm{/}}(\hbar_{\mathrm{e}} t)$, measured following the 1D spreading of a wave packet in momentum space. The error bars represent the typical uncertainty coming from the experimental determination of $\left\langle {p_1^2} \right\rangle$. The different symbols (circles, diamonds and squares) correspond to three sets of different microscopic parameters (*K* and *N*) of the system: (*K*, *N*) ∈ {(4, 3), (4.5, 4), (3.5, 5)} (orthogonal, orange) and respectively (*K*, *N*) ∈ {(2.5, 3), (4, 4), (1.6, 5)} (unitary, blue), for a value of $\hbar_{\mathrm{e}}=1$ . All data in each class collapse onto two distinct universal *β*(*g*) functions, characteristic of each symmetry class, indicated by the shaded regions. The asymptotic behavior at large *g* is correctly predicted by Eq. (6) (continuous lines) inside their domain of validity

Using this prescription, we measured $\left\langle {p_1^2(t)} \right\rangle$ by averaging the experimental momentum distributions over a large number (100) of realizations of these random phases, with the microscopic parameters *K*, *N*, and $\hbar_{\mathrm{e}}$ fixed. In the absence of quantum interference, $\left\langle {p_1^2} \right\rangle$ evolves diffusively with time: $\left\langle {p_1^2} \right\rangle = 2D_0t$. In the orthogonal class, self-intersecting single-loop (CBS-like) interference paths, which are already present from very short times, lead to a rapid deviation from classical diffusion (Fig. 4a). In the unitary class, where the one-loop corrections are absent, this has a dramatic effect on transport properties, leading to a slower deviation from classical diffusion (Fig. 4b).

The leading corrections to $\left\langle {p_1^2(t)} \right\rangle$ due to loops have been calculated for the kicked rotor in ref. ^38^, both in the orthogonal and unitary classes (the unitary-class correction is given with the wrong sign in ref. ^38^—C. Tian, private communication). They allow us to compute the lowest-order correction to the *β*(*g*) function, valid in the limit of large conductivities:

6 $$\begin{array}{*{20}{l}} {\beta (g) = - 1 - \frac{{4\sqrt 2 }}{{3\sqrt \pi g}}} \hfill & : \hfill & {{\mathrm{orthogonal}}{\kern 1pt} {\mathrm{class,}}} \hfill \\ {\beta (g) = - 1 - \frac{1}{{2g^2}}} \hfill & : \hfill & {{\mathrm{unitary}}{\kern 1pt} {\mathrm{class}}{\mathrm{.}}} \hfill \end{array}$$

In order to test these predictions, and the universality of *β*(*g*), we studied a series of different values for the microscopic parameters *K* and *N*, in the two symmetry classes. The measured *β*-functions are shown in Fig. 4c. A remarkable feature of these results is that all data collapse on two distinct scaling functions, as evidenced by the shaded zones, characteristic for each universality class. This constitutes an experimental demonstration of the validity of the one-parameter scaling law. It also shows that the shape of the *β*(*g*) function constitutes a clear marker of the presence or absence of the *PT*-symmetry.

The experimental results in the unitary class are in excellent agreement with (6). This is also true in the orthogonal class, in the limit of large *g* (typically for 1/*g* < 0.5). For smaller values of *g*, we notice deviations from (6), which we confirmed through numerical simulations. This probably indicates that higher-order interference diagrams should be taken into account in the orthogonal class. These observations will hopefully stimulate further theoretical investigations in this direction.

## Discussion

The striking observations reported in the present work highlight the importance of symmetries for the localization and transport properties of disordered media, and the possibility to control them using an artificial gauge field—generated here by appropriately tailoring the driving parameters of a Floquet system. Our method presents a remarkable experimental simplicity, and avoids both the complexity and limitations in more involved schemes (using, e.g., close-to-resonance Raman-dressing of internal states). We characterized the Anderson localization from a different perspective, by directly probing interferential building blocks such as the coherent back- and forward-scattering phenomena. We also measured, in perfectly controlled conditions, the *β*(*g*) scaling function—a universal characteristic measure of transport in disordered media. Moreover, we demonstrated the different sensitivity of these effects with respect to the artificial gauge field flux, which controls the *PT*-symmetry properties of the system.

Interference signatures (such as the CFS) could provide valuable tools to observe the Anderson transition and probe its critical properties in higher dimensions and different symmetry classes. Engineering spin-orbit-coupled dynamical Floquet systems (e.g., using internal-state-dependent optical potentials) would allow, for example, to study the symplectic symmetry class, where the Anderson metal-insulator transition is expected to occur in dimensions as low as two. This also opens an avenue for the study of fascinating phenomena, like quantum Hall effect^39^, Floquet topological insulators and artificial magnetism.

## Methods

### Experiment

In the experiment, we start from a laser-cooled Cesium atomic sample, prepared in a thermal state ($T \simeq 1.5$ μK). The cloud is kicked along the vertical *x* axis by a far-detuned, pulsed (period *T*_1_) optical standing wave (SW), which is created by two independent lasers beams. This allows us to control the amplitude and phase of the potential (via the RF signal sent to two different AOMs) and to shape the modulation sequences ${\cal K}(t)$ and *a*(*t*) as in Eq. (1). The laser parameters are: the detuning Δ = −13 GHz (at the Cs D2 line, wavelength *λ* = 852.2 nm), the waist *w*_0_ = 800 *μ*m and the maximum intensity *I* = 30 W cm^−2^ per beam. The pulse duration is *τ* = 200 ns, while *T*_1_ is typically varied between 10 and 30 μs. After the desired number of kicks, the cloud is allowed to expand and the momentum probability density ${\mathrm{{\Pi}}}(p,t) = \left| {{\mathrm{\Psi }}(p,t)} \right|^2$ is measured using a time-of-flight (TOF) technique^34^.

The TOF expansion time *t* = 170 ms corresponds to a dropping distance of $g t^2/2=14\,{\mathrm {cm}}$ along the vertical *z* direction. The atomic cloud is detected at this location using absorption imaging in a homodyne detection scheme. We utilize a 10 mW probe beam with corresponding waists *w*_*z*_ = 300 μm and *w*_*x*_ = 3.3 mm in the vertical and horizontal directions, respectively. The probe passes through a 45 MHz phase modulator, one of the sidebands being resonant with the atomic transition. The transmitted power of the probe is then detected using a fast photodiode, whose signal is demodulated in real-time using a spectrum analyzer. Residual magnetic field effects are negligible, as the currents in the MOT coils are switched-off at the beginning of the molasses phase, typically 50 ms before starting the kick sequence.

For the CBS/CFS measurements (Section “Coherent Back and Forward Scattering”), it is crucial to utilize a sample with an initial momentum distribution narrower than the width of a Brillouin zone. Indeed, the CBS and CFS peaks have widths given by that of the initial state, and their respective contrasts (equal to one in the ideal case) is strongly reduced otherwise. In order to decrease the mean kinetic energy of the sample, the atoms are loaded in a very shallow 1D optical lattice (vertical direction), whose depth is less than the initial temperature. This filters out the most energetic atoms. Subsequently, we realize 1D adiabatic cooling by switching off the lattice in ~ 1 μs, reaching a momentum distribution width <0.67 × 2*ħk*_*L*_, which corresponds to an equivalent 1D temperature <400 nK (this value is limited by the resolution of the time-of-flight detection). The filtering technique used for studying the CBS and CFS peaks is less suitable for the *β*(*g*) measurements, where starting from a narrow momentum distribution is less crucial whereas having a larger atom number is important to increase the detection signal-to-noise ratio. We thereby used a standard molasses (1.5 μK) as a starting point for these experiments (Section “Symmetry and transport: universal one-parameter scaling law”).

### Units

We have chosen scaled variables in order to express the Hamiltonian in the dimensionless form Eq. (1): distances along the *x* axis are measured in units of (2*k*_*L*_)^−1^ (where *k*_*L*_ is the SW wave number), time in number of kicks (or units of *T*_1_), the particle mass is unity. The Hamiltonian (1) is associated with the Schrödinger equation $i \hbar_{\mathrm{e}}{\partial \psi }/{\partial t} = \hat H\psi$, where $\hbar_{\mathrm{e}} \equiv 4\hbar k_L^2T_1/M$ plays the role of an effective Planck constant, which can be adjusted at will by modifying, e.g., the kick period *T*_1_ (*M* is the Cs atomic mass). The canonical commutation relation reads $[\hat x,\hat p] = i\hbar_{\mathrm{e}}$.

The Hamiltonian (1) is spatially 2*π*-periodic, so that the solutions of the Schrödinger equation can always be expanded on a discrete lattice in momentum space $p_{m}=(m+\beta)\hbar_{\mathrm{e}}$, where *m* is an integer and −1/2 < *β* ≤ 1/2 is the quasimomentum, varying in the first Brillouin zone. Due to the spatial periodicity of the system, *β* is a constant of motion, so that the whole analysis can be performed for *β* = 0.

### Decoherence and choice of experimental Hamiltonians

Decoherence in our setup comes mainly from residual spontaneous emission and fluctuations in the SW phase, and is one of the major limitations of all the experiments presented here. To keep it under control, we use rather small average values of *K*, which somewhat conflicts both with the observation of the CBS and CBS peaks and with precisely measuring the *β*(*g*) scaling function, as explained below.

CBS/CFS peaks from Hamiltonian (5) In the Section corresponding to the CBS/CFS measurements, we use a relatively large (*N* = 10) modulation period, which is suitable for achieving a proper temporal separation between the CBS and CFS peaks. Moreover, in order to properly resolve the CFS dynamics, one also needs a sufficiently large *t*_loc_. For the kicked rotor this is usually obtained by increasing the kick amplitude *K*, which unfortunately decreases the decoherence time *t*_dec_ in the experiment. On the other hand, it turns out that adding a period-two phase modulation increases (for certain values of the phase-shift *a*) the diffusion coefficient *D*_0_, and thus *t*_loc_, without affecting *t*_dec_. This is why, for optimizing the experimental conditions for the measurements of the CBS and CFS dynamics, we opted for the combination of phase and amplitude modulations (5), choosing the value *a* = 0.21 × *π*, found to maximize *D*_0_.

### *β*(*g*) measurements

The scaling function $\beta\equiv {\mathrm{d}}\ln (g)/{\mathrm{d}}\ln (L)$ is extremely sensitive to spurious effects such as experimental noise. In particular, at low values of the kick amplitude *K* (used to keep decoherence under control), short-time correlations between kicks are known to occur, and lead for instance to well-known oscillations in the diffusion coefficient^13,40^). These temporal correlations are responsible for large-amplitude oscillations of $\left\langle {p^2(t)} \right\rangle$, which are magnified when computing the *β*(*g*) function (defined as a logarithmic derivative).

In order to eliminate these effects and properly measure *β*(*g*), it is therefore required to average over as many disorder realizations as possible. For this purpose, the best strategy, as confirmed by numerical simulations, is to average over a large number of realizations of the random phase sequence *a*(*t*). This method was used for our *β*(*g*) measurements: Each experiment is repeated 500 times, with a total of 100 different random realizations of *a*(*t*), and resulting momentum distributions $\left| {{\mathrm{\Psi }}(p,t)} \right|^2$ are averaged. To determine $\left\langle {p^2(t)} \right\rangle$, we fit the measured distribution of squared-momenta $\left| {p{\mathrm{\Psi }}(p,t)} \right|^2$ using the Lobkis-Weaver expression of the momentum distribution^41^.

### Symmetry and times of occurrence of CBS and CFS peaks

The pulse sequence is modulated using a combination of amplitude and phase modulations, as in (1). The kick amplitude sequence ${\cal K}(t)$ has a period of 5, whereas the phase *a*(*t*) is modulated with a period of 2 (represented in Fig. 4 by the different colors used for the even and odd kicks), with an overall period *N* = 10. A consequence of the period-two phase modulation *a*(*t*) is that *PT*-symmetry axes only occur in-between kicks (and never during a kick) which explains why CBS peaks do not occur for odd values of the kick number. A simple analysis of (5) shows that the corresponding Hamiltonian is *PT*-symmetric (belonging thus to the orthogonal class) when the phase $\tilde \varphi \in 2\pi \times \left\{ {0,\frac{1}{5},\frac{2}{5},\frac{3}{5},\frac{4}{5}} \right\}$. Each of these values of the $\tilde \varphi$ leads to a different time of occurrence of the CBS peak—corresponding to kicks {6, 10, 4, 8, 2}, respectively.

**Fig. 5:**
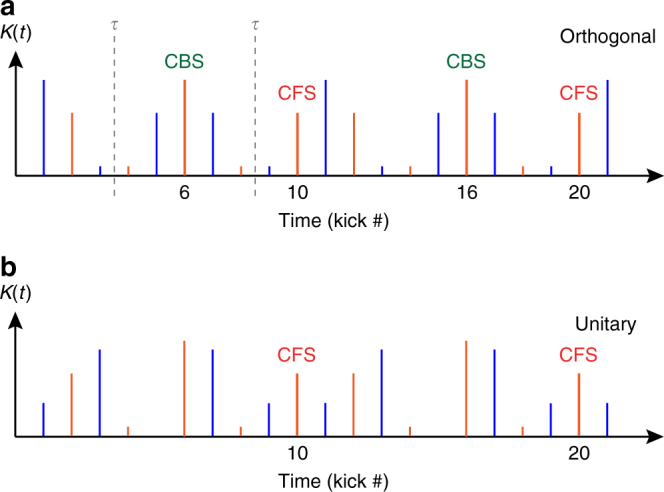
Two pulse sequences belonging to different symmetry classes. The sequences correspond to the data shown in Fig. 2, and were obtained using two different values of the symmetry-control parameter $\tilde \varphi$ in (5): $\tilde \varphi = 0$ (**a**) and $\tilde \varphi = - 3\pi {\mathrm{/}}5$ (**b**), for which the system belongs respectively to the orthogonal and unitary symmetry class. In the orthogonal class the time sequence has symmetry axes *τ*; a CBS peak will appear at the kicks symmetric to the initial kick with respect to such axis. In the unitary class no CBS peak will exist. In both cases, the symmetry-insensitive CFS peaks are expected to occur at integer multiples of the period (*N* = 10)

Take for instance the modulation sequence shown in Fig. 5, corresponding to experimental data in Fig. 2. When $\tilde \varphi = 0$ (Fig. 5a), the sequence has *PT*-symmetry axes (vertical dashed lines labeled, *τ*), and the system belongs to the orthogonal class. In this case, CBS peaks are expected to appear periodically, at kicks 6 (mod. 10), i.e., at times equal to twice the occurrence time of each *τ*. On the other hand, when $\tilde \varphi \, \notin \, 2\pi \times \left\{ {0,\frac{1}{5},\frac{2}{5},\frac{3}{5},\frac{4}{5}} \right\}$ no symmetry axes exist (e.g. in (Fig. 5b), for $\tilde \varphi = - 3\pi /5$). In both universality classes the symmetry-insensitive CFS peaks occur at integer multiples of the period of the system, i.e., at kicks 0 (mod. 10).

### The CBS and CFS contrast measurements

Analyzing the experimental data in Fig. 2, we can extract the contrasts *C*_*B*_(*t*) and *C*_*F*_(*t*), of the CBS and CFS peaks respectively, vs. time. The contrasts, for either case, are defined as: *C*_*B*,*F*_(*t*) = (Π_0_(*t*) − Π_0,incoh._(*t*))/Π_0,incoh._(*t*), and are evaluated at the occurrence times of their respective peaks, *t*_CBS_ and *t*_CFS_ (corresponding respectively to red and and green points in Fig. 2). Here, ${\mathrm{{\Pi}}}_0(t) = \left| {{\mathrm{\Psi }}(p_1 = 0,t)} \right|^2$ is the total zero-momentum probability density (also defined in the main text), while Π_0,incoh._(*t*) corresponds to the incoherent (classical) contribution to Π_0_(*t*). Outside *t*_CBS_ and *t*_CFS_ (i.e., at times corresponding to the black points in Fig. 2), the two contributions are identical: Π_0_(*t*) = Π_0,incoh._(*t*). In order to evaluate *C*_*B*,*F*_(*t*), we interpolate the Π_0,incoh_.(*t*) values at *t*_CBS_ and *t*_CFS_. This method was used for the data shown in Fig. 3.

### Data availability

The data that support the findings of this study are available from the corresponding author upon request.
